# Prediction of bleeding risk in patients taking vitamin K antagonists using thrombin generation testing

**DOI:** 10.1371/journal.pone.0176967

**Published:** 2017-05-04

**Authors:** Saartje Bloemen, Suzanne Zwaveling, Hugo ten Cate, Arina ten Cate-Hoek, Bas de Laat

**Affiliations:** 1 Synapse Research Institute, Cardiovascular Research Institute Maastricht, Maastricht University Medical Center, Maastricht, the Netherlands; 2 Department of Biochemistry, Cardiovascular Research Institute Maastricht, Maastricht University Medical Center, Maastricht, the Netherlands; 3 Laboratory for Clinical Thrombosis and Hemostasis, Department of Internal Medicine, Cardiovascular Research Institute Maastricht, Maastricht University Medical Center, Maastricht, the Netherlands; Universita degli Studi Magna Graecia di Catanzaro, ITALY

## Abstract

Until recently, vitamin K antagonists (VKAs) were the mainstay of oral anticoagulant treatment with bleeding as the most prevalent adverse effect. One to four percent of patients experience major bleeding episodes, while clinically relevant bleeding occurs in up to 20%. At this moment no laboratory assays are available to identify patients at risk for bleeding. With this study we aimed to investigate whether thrombin generation tests might identify a bleeding risk in patients taking VKAs. This prospective cohort study included 129 patients taking VKAs for more than three months. Calibrated automated thrombinography (CAT) was performed in whole blood, platelet rich and platelet poor plasma. Hematocrit, hemoglobin concentrations and the International Normalized Ratio (INR) were defined and coagulation factor levels were measured. Forty clinically relevant bleeding episodes were registered in 26 patients during follow-up. No differences were found in plasma CAT parameters or INR values. Bleeding was not associated with age, sex, hematocrit, hemoglobin levels or coagulation factor levels. In whole blood a significantly lower endogenous thrombin potential (ETP) and peak were found in patients with bleeding (median ETP: 182.5 versus 256.2 nM.min, p = 0.002; peak: 23.9 versus 39.1 nM, p = 0.029). Additionally, the area under the receiver operating curve (AUC ROC) was significantly associated with bleeding (ETP: 0.700, p = 0.002; peak: 0.642, p = 0.029). HAS-BLED scores were also significantly higher in bleeding patients (3 versus 2, p = 0.003), with an AUC ROC 0.682 (p = 0.004). In conclusion, bleeding in patients taking VKAs is associated with a decreased whole blood ETP and peak as well as with an increased HAS-BLED score.

## Introduction

For over 50 years vitamin K antagonists (VKAs) have been widely used, not only as (first choice) treatment for thromboembolism, but as primary and secondary prevention of (venous) thromboembolism as well [1]. Warfarin is currently the most prescribed VKA followed by acenocoumarol and phenprocoumon. The predominant adverse effect of anticoagulant therapy is an increased risk of bleeding which can lead to morbidity and mortality. Annually approximately 1 to 4% of patients treated with VKAs suffer from major bleeding episodes [2]. Clinically relevant bleeding occurs in up to 20% of patients [3]. The risk of bleeding increases with age. Patients that are older than 75 years, experience major bleeding more frequently than younger patients: 5.1% versus 1% per year, respectively [4]. This bleeding risk increases even more when VKAs are combined with antiplatelet therapy [5].

In the past several attempts were made to more accurately estimate the bleeding risk of individual patients treated with VKAs. One of the commonly used clinical methods for the identification of patients with atrial fibrillation at risk for bleeding is the HAS-BLED score, which is a clinical decision score [6]. The HAS-BLED score contains the risk factors hypertension, abnormal renal/liver function, stroke, bleeding history or predisposition, labile international normalized ratio (INR), elderly (age ≥ 65 years) and drugs/alcohol (ab)use concomitantly. Although the HAS-BLED score is developed and validated only in patients with AF, it would be reasonable to think that the score could be applied in patients with different indications for VKA use, considering the comparable risk factors for bleeding. Moreover, the HAS-BLED score has the highest predictive potential compared to other clinical prediction scores [7]; however its accuracy differed based on the cohort used for validation [8, 9]. As of yet there are no laboratory methods that prospectively predict which patients are at risk for bleeding. Considering the INR, there is an increased risk of bleeding at higher INR levels, yet the majority of bleeding events occurs in patients that are within the therapeutic range.

Thrombin generation, a method that detects the enzymatic activity of thrombin, has been shown to be able to detect both prothrombotic and bleeding phenotypes based on changes in the coagulation system [10]. Additionally, thrombin generation has the capacity to detect the anticoagulant effect of many if not all anticoagulants, including VKAs and direct oral anticoagulants (DOACs) [11, 12]. Until recently this method was only applicable in plasma due to quenching of the fluorescent signal by sedimentation of erythrocytes. Introduction of a porous matrix, preventing this sedimentation, and using a different thrombin-sensitive substrate enabled studying thrombin generation in whole blood [13]. In this study we investigated whether thrombin generation, in plasma or whole blood, could be used to predict bleeding episodes in 129 patients taking VKAs and compared these parameters to the INR, the HAS-BLED score, fibrinogen levels and other factor determinations.

## Materials & methods

### Study population

Patients taking VKAs were randomly included in this study between March 2012 and October 2013. A sample size of 127 was sufficient to provide power of 80% with a two-sided α-level of 0.05. Patients were eligible for inclusion in this study when treated with VKAs for longer than three months and undergoing a venapuncture in order to determine their INR value at the Maastricht anticoagulation clinic. Patients under 18 years of age were excluded. All patients were informed and provided written consent. The study was approved by the local medical ethical committee (Medisch-ethische toetsingscommissie academisch ziekenhuis Maastricht/universiteit Maastricht (METC aZM/UM), approval number: 11-4-142.4/ccl).

### Blood samples

Patients were included in the anticoagulation clinic during one of their routine checkups for VKA treatment. After giving informed consent on this day of inclusion, blood was drawn for both INR determination and further laboratory assays necessary for this study, such as thrombin generation. Blood was collected through antecubital venapuncture using citrate tubes (1 volume of trisodium citrate 3.2% to 9 volumes of blood), (BD Vacutainer system, Roborough, Plymouth, UK). Platelet rich plasma (PRP) was prepared by centrifuging the blood at 250g for 15 min. Platelet poor plasma (PPP) was prepared by double centrifugation at 2,000g for 10 min. PPP for thrombin generation was used immediately, the remainder was stored at -80°C until bulk analysis in other assays was possible.

### Follow-up

Bleeding episodes were recorded during the follow-up period (minimum four months, mean follow-up time: 15.5 months) at the anticoagulation clinic in Maastricht according to the definitions and criteria defined by the Dutch Federation of Anticoagulation Clinics. These criteria are based on the guidelines by the Scientific and Standardization Committee of the International Society on Thrombosis and Haemostasis [14]. The definitions of major bleeding are: any intracranial hemorrhage, any objectively diagnosed intra-articular hemorrhage and bleeding leading to death, transfusion, surgery, and/or hospital admission. Minor bleeding was defined as all other clinically relevant bleeding not meeting the definition for major bleeding. Clinically relevant bleeding was defined according to the guidelines of the Dutch Federation of Anticoagulation Clinics. Bleeding events, when detected by a general practitioner or at the hospital, were systematically reported to the anticoagulation clinic. Minor bleedings were mostly reported by patients themselves, during routine visits and only rated if they were clinically relevant. The specifications of the types of bleeding (minor/major) in our study are listed in Table 1.

**Table 1:** Bleeding types with specifications and the number of each type of bleeding that occurred during the follow-up period.

Type	Specification	Classification	Number of bleedings
Nose	> 30 min	Minor	6
Eye	conjunctival	Minor	12
Skin	> 10 cm or multiple hematomas	Minor	7
Digestive tract		Minor	3
Uro-genital tract		Minor	5
Traumatic bleed		Minor	2
Nose	> 30 min, with treatment	Major	2
Digestive tract		Major	2
Other locations		Minor	1

We also recorded age, sex, type of VKA, time in therapeutic range (TTR) and indication for VKA use. The use of antithrombotic therapy as well as other co-medications affecting coagulation (e.g. non-steroidal anti-inflammatory drugs (NSAIDs)) was also documented to assess potential confounding effects. Bleeding episodes were recorded until the end of the study or until discontinuation of treatment. Other parameters were determined on the day of inclusion.

### Reagents

Synthetic phospholipids were obtained from Avanti Polar Lipids Inc. (Alabaster, AL, USA). Recombinant tissue factor (TF) known as Innovin (Dade-Behring, Marburg, Germany) was used. Z-Gly-Gly-Arg-aminomethylcoumarine (ZGGR-AMC) was purchased from Bachem (Basel, Switzerland). Rhodamine substrate (P_2_Rho) was a gift of Diagnostica Stago (Asnières sur Seine, France). Recombinant human thrombomodulin (TM) was a kind gift of Asahi Kasei Pharma (Japan). The calibrator, α2-macroglobulin-thrombin complex, was prepared as described previously [15]. Hepes buffers containing 5 mg/ml or 60 mg/ml bovine serum albumin were prepared as described by Hemker et al. [16].

### Thrombin generation

CAT was performed in plasma as described earlier [15]. TF was used to initiate the reaction at a final concentration of 1 pM in PRP and at both 1 pM and 5 pM with 4 μM phospholipids in PPP. The effect of TM was also tested in PPP (1 pM TF) at a concentration of 2.5 nM. In PPP (5 pM TF) and PRP 20 nM of TM was used (around IC_50_). The whole blood CAT technique was performed according to our group’s earlier specifications [13]. Thirty microliters of blood were mixed with 10 μl of P2Rho substrate (1.8 mM) and 20 μl of TF/CaCl_2_ solution were added, which initiated thrombin generation. In the calibration wells, 20 μl of reagents were replaced with calibrator (final concentration: 100 nM). Instantly after the activation, 5 μl of the mixture were pipetted on paper disks (Whatman 589/1, Whatman GmbH, Dassel, Germany) in a flat bottom 96-well polystyrene plate and covered with 40 μl of mineral oil (Affymetrix, USB, Cleveland, Ohio, USA). The final TF concentration was 1 pM and TM (20 nM) was added as well. Fluorescent signals were measured using the Fluoroskan Ascent software (Thermo Labsystems, Helsinki, Finland). Measurements were performed in triplicate and fluorescent signals were transformed into thrombin concentrations as described by Hemker et al. [17]. The thrombin generation parameters which were analyzed were: lag time, which is the time until the first traces of thrombin are formed; ETP, the area under the thrombin generation curve, peak level of thrombin formation and the time-to-peak or the time until the thrombin peak is reached.

### Additional analyses

Hematocrit and hemoglobin concentration determinations were performed in citrated blood with a Coulter Counter analyzer (Beckman Coulter, Woerden, the Netherlands). Levels of clotting factors, including fibrinogen were assessed using the STA-R Evolution analyzer (Stago, Asnières sur Seine, France). Factor II, V, VII, VIII, IX, X levels were determined with clotting assays triggered by either a thromboplastin based reagent (FII, FV, FVII, FX) or a kaolin based reagent (FVIII and FIX). Fibrinogen levels were measured using the Clauss method. Protein C activity was determined by an aPTT based assay, activated by Agkistrodon c. contortrix venom. Protein S was tested in a clotting assay in which the activity of protein S as a cofactor of protein C is measured by its effect on factor Va. Antithrombin (ATIII) was determined by a chromogenic measurement. INRs were determined in the local anticoagulation clinic. The prothrombin time was determined in citrated plasma with an automated coagulation analyzer (Sysmex CA 1500, Siemens Diagnostics, the Netherlands) using Innovin® (Dade-Behring) as the thromboplastin reagent. The INR value was expressed as the ratio of the subject’s PT to a normal (control) sample raised to the power of the International Sensitivity Index (ISI); (PT_test_/PT_normal_)^ISI^. HAS-BLED scores of each patient were calculated post-hoc by a blinded physician using medical records, allotting one point for each risk factor [18].

### Statistical analysis

All analyses were performed using Graphpad Prism version 5.00 (Graphpad Software Inc., La Jolla, CA, USA). Patients with missing data were not excluded from the analysis. Correlation analysis of whole blood versus plasma CAT parameters was performed using the Pearson correlation test. Patients were divided into two groups (with bleeding and without bleeding). Differences between groups were analyzed via the Mann Whitney U test and represented by medians with interquartile ranges (IQR), range from minimum value to maximum value and 95% confidence intervals (CI). Receiver operating curves (ROC) were used to investigate the ability of WB CAT and the HAS-BLED score to discriminate between bleeding and non-bleeding patients. The area under the ROC curve (95% CI) quantified the predictive value of parameters. Differences between two groups (other than bleeding versus non-bleeding) were also analyzed using the Mann Whitney U test. A two-sided p-value of ≤ 0.05 was considered statistically significant.

## Results

### Patient characteristics

One hundred and fifty patients were eligible for the study and 21 patients had to be excluded for several reasons: failed blood collection, not fulfilling the inclusion criterion of using VKA for at least three months, technical problems during measurements or other reasons. The demographics of the remaining 129 patients are listed in Table 2. The average duration of VKA treatment until the inclusion date was approximately 5 years. Therapeutic ranges consisted of INR’s from 2.0–3.5 (n = 103) or 2.5–4.0 (n = 26), depending on the indication.

**Table 2:** Patient demographics and clinical characteristics.

*Demographics*				
* *	*Total population (n = 129)*	*Non-bleeding (n = 103)*	*Bleeding* *(n = 26)*	*p-value*
**Age** (median [IQR])	70 [62.5–76.0]	70 [60–80]	70 [60–80]	0.4074
**Female sex** (%)	22	21	23	0.8532
**VKA** Acenocoumarol (%)	95	96.1	92.3	0.4163
**Patients with TTR<60%** (%)	80	84.5	61.5	0.0214
**Indications** (%)				
*AF*	72.1	73.8	65.4	
*prosthetic valve*	13.2	10.7	23.1	
*pulmonary embolism*	3.9	3.9	3.8	
*venous thrombosis*	3.1	3.9	0	
*CABG*	1.6	1.9	0	
*peripheral atherosclerosis*	1.6	1.0	3.8	
*cardiomyopathy*	0.8	1.0	0	
*cerebrovascular insufficiency*	0.8	1.0	0	
*cerebral embolism*	0.8	1.0	0	
*arterial embolism*	0.8	1.0	0	
*other rare indications*	1.6	1.0	3.8	

SD, standard deviation; VKA, vitamin K antagonist; AF, atrial fibrillation; CABG, coronary artery bypass graft

### Bleeding episodes

In our study we found that 26 patients (20.2%) suffered from 40 clinically relevant bleeding episodes during a mean follow-up of 15.5 months after inclusion. The mean time between inclusion (including testing) and bleeding was 9.8 months. The bleeding rates in male (n = 121) and female (n = 28) patients were comparable (20% and 21% bleeding, respectively). Seventeen patients had one bleeding episode, six patients experienced two bleeding events, two patients had three bleeding episodes and one patient suffered five times from a clinically relevant bleeding. Patients experienced different types of bleeding (Table 1). In our population patients mainly experienced conjunctival eye bleeds. Four major bleeding episodes occurred during the follow-up period. Two patients experienced severe digestive tract bleedings and one patient suffered twice from severe nosebleeds.

### Thrombin generation

**Fig 1:**
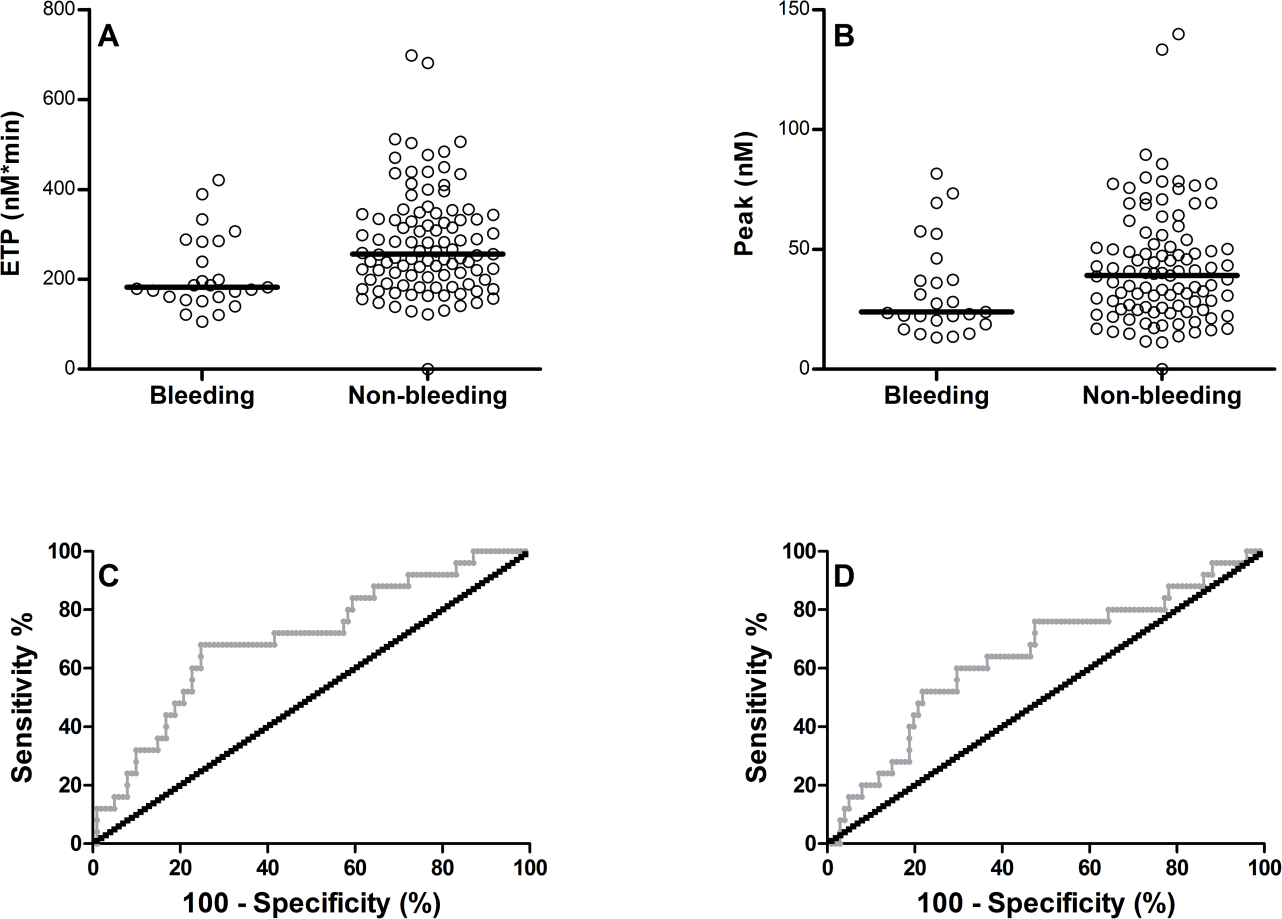
Whole blood CAT analysis of patients with and without bleeding symptoms. (A) Difference in whole blood endogenous thrombin potential (ETP) (p = 0.002) in patients with and without bleeding. (B) Difference in whole blood peak (p = 0.029) in patients with and without bleeding. (C) Receiver operating curve (ROC) of the ETP in whole blood thrombin generation (area under the curve (AUC) = 0.700, p = 0.002). (D) Receiver operating curve (ROC) of the peak in whole blood thrombin generation (AUC = 0.642, p = 0.029).

Analyzing samples with whole blood CAT a significantly lower ETP and peak was found in the patients that suffered from bleeding compared to patients that did not have this adverse effect (Fig 1A and 1B and Table 3). Differences in ETP and peak remained statistically significant in the presence of TM, although to a lesser degree (median [IQR] (CI) ETP: 134.9 [104.7–193] (126.4–169.9) versus 174.3 [129.5–222.9] (169.0–195.7), p = 0.009; peak: 20.18 [13.74–30.39] (18.0–28.9) versus 27.72 [18.08–37.49] (27.1–37.0), p = 0.033 (n = 25 and n = 102)), in bleeding versus non-bleeding patients respectively). The lag time and time-to-peak did not differ significantly between bleeding and non-bleeding patients. A receiver operating curve (ROC) was constructed for ETP and peak determined in whole blood (Fig 1C and 1D). Assessment of the area under the curve (AUC) of the ROC demonstrated that both ETP and peak were significantly associated (AUC (CI) ETP: 0.700 (0.584–0.816), p = 0.002 (n = 25) and AUC (CI) peak: 0.642 (0.516–0.767), p = 0.029 (n = 102), respectively) with the bleeding tendency.

**Table 3:** Medians with interquartile ranges of thrombin generation parameters in plasma.

		*Non-bleeding*					*Bleeding*					* *
		*n*	*median*	*IQR*	*min-max*	*CI*	*n*	*median*	*IQR*	*min-max*	*CI*	*p-value*
**Whole blood**	*ETP (nM*.*min)*	101	256.2	194.9–344.2	0–698.0	258.5–304.5	25	182.5	157.2–284.7	106.0–420.5	178.1–247.3	*0*.*002*
	*Peak (nM)*	101	39.1	24.9–53.2	0–140.0	38.1–47.5	25	23.9	19.6–41.8	13.3–81.7	25.1–41.5	*0*.*029*
	*Lag time (min)*	101	6.5	5.4–7.6	0–14.7	6.2–7.1	25	7.1	5.2–8.0	3.5–12.6	6.0–7.9	*0*.*545*
	*Time-to-peak (min)*	101	12.2	9.8–14.9	0–222.3	10.4–18.7	25	12.2	9.6–17.5	6.7–23.3	11.7–15.5	*0*.*427*
**PRP**	*ETP (nM*.*min)*	101	323.0	208.8–406.5	0–817.0	294.8–361.4	23	291.5	226.0–374.5	0–532.5	247.4–357.9	*0*.*510*
	*Peak (nM)*	101	23.1	14.8–33.7	0–71.4	22.3–28.4	24	20.1	14.0–28.3	0–52.8	17.5–26.5	*0*.*521*
	*Lag time (min)*	101	18.0	12.7–24.1	0–102.5	17.9–23.4	24	18.4	13.3–37.3	7.3–62.1	18.6–31.7	*0*.*273*
	*Time-to-peak (min)*	101	30.0	22.8–39.7	0–107.3	30.0–36.0	24	29.1	23.8–53.3	18.3–81.9	31.2–47.6	*0*.*236*
**PPP (5 pM TF)**	*ETP (nM*.*min)*	102	437.5	343.9–540.9	139.5–1304.	427.3–510.9	26	367.3	298.4–501.4	116.5–1012	330.9–476.0	*0*.*094*
	*Peak (nM)*	103	86.6	62.9–114.3	26.4–253.4	86.1–102.6	26	76.2	59.0–98.9	23.8–202.5	67.9–98.1	*0*.*181*
	*Lag time (min)*	103	5.5	4.0–6.8	2.5–18.3	5.3–6.3	26	5.1	3.9–7.4	2.0–16.3	4.9–7.6	*0*.*805*
	*Time-to-peak (min)*	103	8.3	6.8–9.5	4.5–21.0	8.0–9.0	26	7.8	6.4–10.1	4.3–18.8	7.5–10.2	*0*.*897*
**PPP (1 pM TF)**	*ETP (nM*.*min)*	103	278.5	181.5–380.0	0–909.0	268.9–340.1	25	227.0	177.5–364.3	0–606.0	206.0–321.8	*0*.*422*
	*Peak (nM)*	103	52.7	29.9–76.3	0–200.0	50.4–66.2	26	42.2	27.7–69.2	0–111.7	35.2–58.1	*0*.*245*
	*Lag time (min)*	103	16.0	11.0–20.5	0–38.5	14.6–17.6	26	15.3	10.4–24.9	0–77.0	13.3–25.3	*0*.*668*
	*Time-to-peak (min)*	103	19.0	14.0–23.8	0–43.5	17.6–20.8	26	19.0	13.4–28.9	0–80.8	16.5–28.8	*0*.*666*

IQR, interquartile range; min, minimum; max, maximum; CI, confidence interval; PRP, platelet rich plasma; PPP, platelet poor plasma; TF, tissue factor

**Fig 2:**
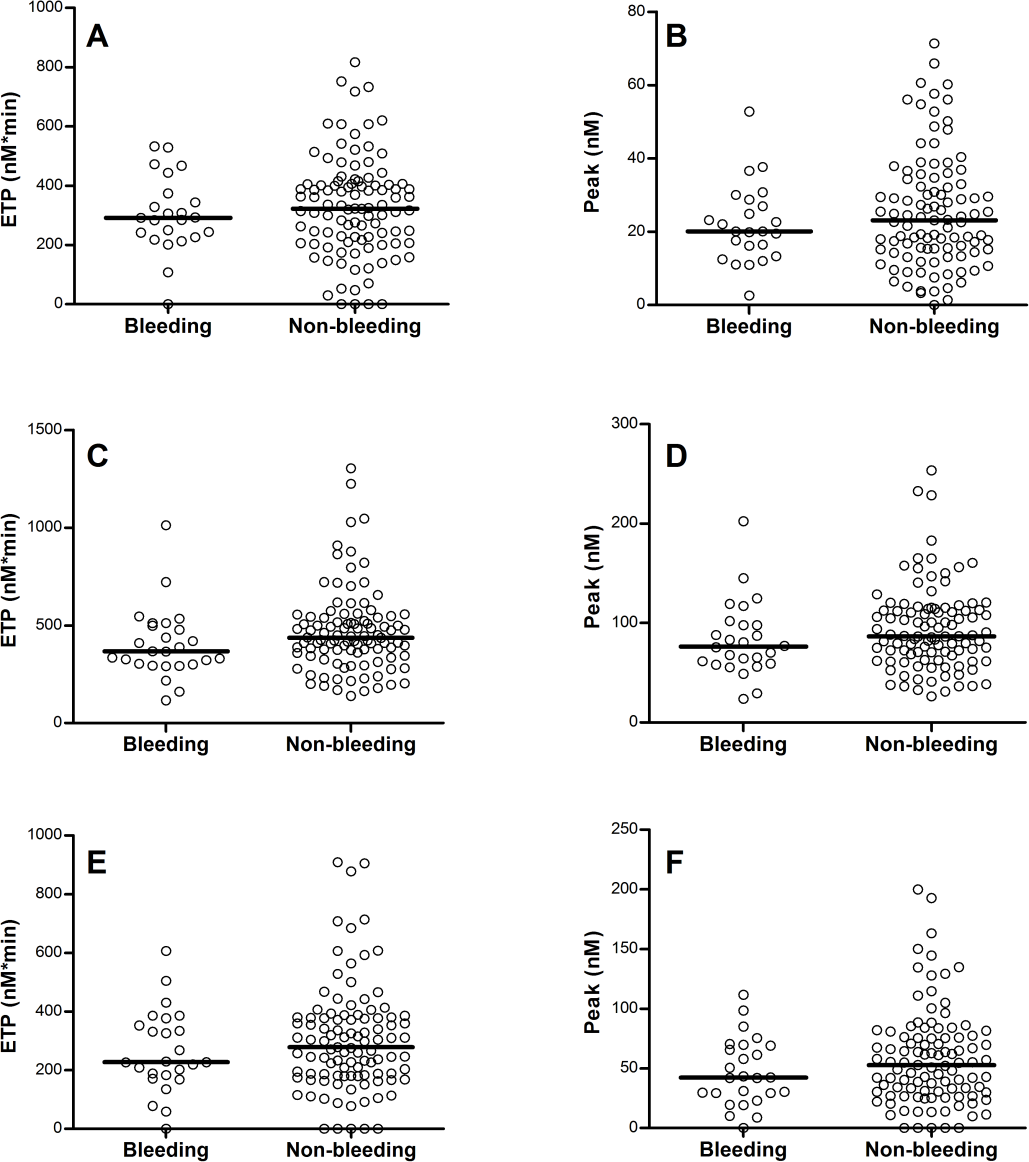
Plasma CAT analysis of patients with and without bleeding symptoms. Analysis of the endogenous thrombin potential (ETP) and peak in patients with and without bleeding. (A) ETP in platelet rich plasma, (B) peak in platelet rich plasma, (C) ETP in platelet poor plasma at 5 pM TF, (D) peak in platelet poor plasma at 5 pM TF, (E) ETP in platelet poor plasma at 1 pM TF, (F) peak in platelet poor plasma at 1 pM TF. No significant differences were found for these parameters using the Mann Whitney U test. Medians are indicated by lines.

In plasma no significant differences were detected for the thrombin generation parameters between patients that suffered from bleeding and those without bleeding events, although a trend for a decreased ETP and peak height was observed in the bleeding population (Fig 2 and Table 3). Similarly, no significant differences were found in the lag time and time-to-peak between both populations. The addition of thrombomodulin did not change these results.

We assessed whether co-medication affecting platelets could have an effect on the ETP and peak in whole blood. Twenty-nine patients used co-medication that affects platelets (P2Y12-inhibitors, acetylsalicylic acid and/or non-steroidal anti-inflammatory drugs) of which 12 (41.4%) suffered from bleeding and 17 patients did not (S1 Table). From S1 Table it can also be calculated that 13.4% displayed bleeding when not using co-medication. We found that the use of these co-medications did not influence the ETP and peak measured in whole blood (S1 Table). Therefore our analyses were not corrected for this type of co-medication.

The parameters in whole blood CAT (1 pM TF) were compared to the parameters (ETP, peak, lag time and time-to-peak) in the plasma CAT (PRP at 1 pM TF and PPP at 1 and 5 pM TF). All thrombin generation parameters displayed a significant correlation between plasma and whole blood measurements and the highest correlation coefficients were established for the ETP and peak (S2 Table). The best correlation for the four parameters was found in PPP using 5 pM TF. Regarding the INR, similar correlations were observed with the whole blood CAT as compared to plasma CAT (inverse, hyperbolical correlation of ETP and peak with INR, linear correlation of lag time and time-to-peak with INR) (S3 Table).

### Additional analyses

Bleeding events were not associated with a difference in age (median [IQR]: 70 [60–80] for both groups (CI bleeding: 60–70 versus non-bleeding: 70–70), p = 0.4074 (n = 26 and n = 103)). Patients with and without bleeding events did not significantly differ regarding the INR, hematocrit, hemoglobin levels, or fibrinogen concentration (S1 Supporting information). Differences in levels of other coagulation and anticoagulant factors were determined, but none of them reached statistical significance (S4 Table).

### HAS-BLED

**Fig 3:**
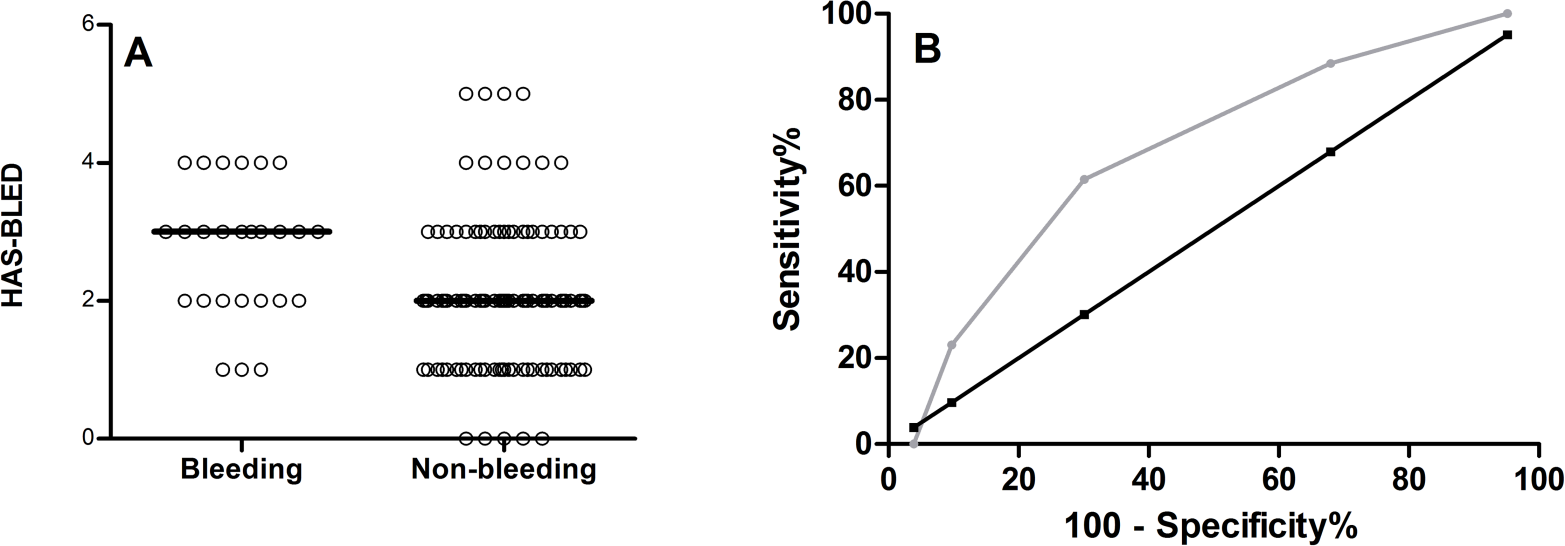
HAS-BLED analysis of bleeding and non-bleeding patients. (A) Difference in HAS-BLED scores of patients with (n = 26) and without bleeding (n = 103, p = 0.003). (B) Receiver operating curve (ROC) of the HAS-BLED scores (area under the curve = 0.682, p = 0.004).

HAS-BLED scores in patients with bleeding episodes were significantly higher than in patients that did not bleed (median [IQR] (CI): 3 [2–3.25] (2.3–3.1) versus 2 [1–3] (1.9–2.3), p = 0.003 (n = 26 and n = 103)) (Fig 3A). A ROC curve was constructed resulting in an AUC of 0.682 (CI: 0.571–0.792) (p = 0.004) (Fig 3B). Additionally, the HAS-BLED score did not correlate to the whole blood ETP (r = 0.051, p = 0.573) and peak (r = 0.075, p = 0.401).

## Discussion

Thrombin generation tests in plasma have been shown to provide a more complete overview of coagulation, since it encompasses both the extrinsic and intrinsic pathway. Clotting times (e.g. PT, aPTT) only include one of the pathways and measure the time until fibrin is formed, representing just 5% of the thrombin formation [11]. It is known that an assessment of the complete thrombin generation is better related to clinical outcomes [10, 19–22]. More recently, the whole blood CAT was developed, bringing thrombin generation one step closer to physiology paving the way for point of care thrombin generation assays. In this study, we provide a first clinical validation of this technique in patients using VKAs. Patients with and without clinically relevant bleeding episodes significantly differed in whole blood CAT ETP and peak. As expected, the INR, which is the standard follow up test for patients taking VKAs, was not predictive for bleeding on the long term; since the treatment of patients is adjusted to the measured INR.

In a population of patients using VKAs the whole blood CAT suggests to be the first laboratory test that is related to a bleeding risk in a prospective set-up. The AUC of ROC is a measure for the discrimination of a test/score between patient with and without the disease. The ETP and peak in whole blood have an AUC of ROC of 0.700 and 0.642, respectively and therefore particularly the ETP can be considered as a fair predictor of bleeding. Although these parameters cannot fully discriminate between bleeding and non-bleeding patients, their discriminative value is at least comparable to that of the HASBLED score. In contrast, the plasma-based CAT did not discriminate between bleeding and non-bleeding patients. Other studies showed that plasma CAT was indicative of bleeding in different patient populations [19, 23, 24]. However, this finding could not be repeated in our study. The presence of platelets alone (PRP) did not improve the outcome of the test considering discrimination between patients with and without bleeding. This is in accordance to earlier findings indicating that recurrent bleeding in patients with a stable INR cannot be explained by changes in platelets or von Willebrand factor function [25]. In contrast to plasma CAT, whole blood CAT includes erythrocytes that can directly contribute to thrombin generation, e.g. as a source of procoagulant phospholipids on the cell membrane. Interplay between the coagulation system and these red blood cells may provide an explanation that whole blood CAT, but not plasma CAT enables discrimination between bleeding and non-bleeding patients in patients on VKA. Earlier studies by Whelihan et al. show that a sub-fraction of red blood cells express phosphatidylserine and this might serve as a surface for thrombin and other phospholipid-bound coagulation factors [26]. Apart from erythrocytes, whole blood differs from plasma in the presence of leukocytes. The release of TF by these cells (in particular monocytes) may also contribute to the differential activation of coagulation in bleeding versus non-bleeding patients. However, the exact mechanism resulting in an improved discrimination of whole blood thrombin generation between patients with and without bleeding episodes needs further research.

Bleedings that occurred during the study were mostly spontaneous, minor bleedings; none were caused by surgical intervention or major trauma. Although this increases the likelihood of similarity in etiology, the bleedings in this study population can still result from multiple factors, e.g. VKA treatment, co-medication, the coagulation system (e.g. clotting factors), age, gender or TTR. Co-medication is one of the known factors which can increase the risk of bleeding [5, 27]. In our population we found that 41.4% of the patients who were taking co-medication affecting platelets suffered from bleeding, whereas this was only 13.4% in patients that did not use co-medication. In this study whole blood ETP and peak were not influenced by the use of co-medication. This confirms previously reported results suggesting that the presence or absence of platelets and by extension platelet agonists or antagonists, had no effect on thrombin generation in whole blood [13]. Moreover, we detected no differences between bleeding and non-bleeding patients in PRP. Therefore our analyses were not corrected for the use of co-medications. Furthermore, clotting factor deficiencies or increased levels of natural anticoagulants could result in an increased bleeding risk. However, both clotting factor and anticoagulant factor levels proved to be similar in the bleeding and non-bleeding patients. Additionally, an increased age or difference in gender distribution may confer an increased risk for bleeding [4, 28, 29]. Yet in our study no significant difference in age or gender distribution was found between the patients with and without bleeding episodes. The TTR (which is also included in the HAS-BLED score) was lower in patients with bleeding. It is known that patients with a low TTR are at higher risk of complications (either bleeding or thrombosis) [30]. A significantly higher HAS-BLED score was detected in patients with bleeding events compared to those without. The ROC AUC of the HAS-BLED score was comparable to that of the whole blood ETP and peak. The HAS-BLED score was developed for AF patients; however other patient groups taking VKAs have comparable risk factors for bleeding. In the present study 72% of the population was treated for AF and 28% of the patients had other indications. Even within the latter group, in spite of its small number, a significant difference in HAS-BLED scores was found between the bleeding and the non-bleeding group, indicating that the HAS-BLED could be of value in these patients. Since bleeding may be provoked by many different factors, it seems unlikely that one single test would be able to predict a patients bleeding risk. Within this reasoning, a combination of laboratory tests with existing bleeding scores, may lead to a more accurate prediction of the bleeding risk. On the other hand, it could also be suggested to include the whole blood CAT ETP and peak in the HAS-BLED score, since this was the coagulation assay with the best clinical association. The INR, which is currently incorporated in the HAS-BLED score, did not prospectively discriminate between bleeding and non-bleeding patients. Additionally, there was no correlation between whole blood TG parameters and the HAS-BLED score. Therefore, we speculate that the predictive value of the HAS-BLED could be improved by replacing the INR by whole blood CAT parameters. This would then open the possibility to also use this score in assessing the bleeding risk for patients using DOACs. We expect this might be important in the future with the increasing use of DOACs.

Inevitably, this study had some limitations. Firstly, the observed risk associations are with all clinically relevant bleeding complications, whereas it could be argued that only major bleeding complications matter. Previous studies showed that patients with minor bleeding events are at increased risk (>2.5-fold) for major bleedings [31, 32]. Therefore, we believe that the inclusion of minor, clinically relevant bleedings is a rational and clinically relevant choice.

Secondly, it can be argued whether this study featured a large enough sample size to discover a relevant effect of bleeding predisposition on thrombin generation, but in accordance with our power calculation, the observed rate of bleeding complications allows for drawing conclusions based on the presented data. It is obvious that in order to assess the utility of whole blood CAT testing in patients on anticoagulation a focus on major bleeding complications will be important, which warrants repetition in a larger study to corroborate our findings.

Finally, it is known that some biomarkers and risk factors associated with bleeding have also been linked to stroke and systemic thromboembolism. Therefore, it would be interesting to explore this topic. Unfortunately we were unable to do so because there were no episodes of recurrent VTE or stroke during the follow-up period.

In conclusion, clinically relevant bleeding in patients taking VKAs in our study is associated with a diminished whole blood ETP and peak, but not with INR. Consequently, whole blood ETP and peak could be of value in assessing patients who suffer from recurrent bleeding with an INR in therapeutic range. In our population an augmented HAS-BLED score was associated with bleeding as well. Implementing whole blood CAT might improve the sensitivity of bleeding scores such as the HAS-BLED, enabling further tailoring of therapy for individual patients.

## Supporting information

S1 Table The use of co-medications did not influence whole blood ETP and peak. (DOCX)

S2 Table Correlation of whole blood CAT with plasma CAT. (DOCX)

S3 Table Correlation of INR with CAT parameters. (DOCX)

S4 Table Medians with interquartile ranges of coagulation factor determinations. (DOCX)

S1 Supporting information Analysis of parameters which are known to be related with bleeding. (DOCX)
