# Supplementary material for: Prediction of bleeding risk in patients taking vitamin K antagonists using thrombin generation testing
Source: PLoS One. 2017 May 4;12(5):e0176967. doi: 10.1371/journal.pone.0176967 (PMC5417600; doi:10.1371/journal.pone.0176967)
Supplement: S1 Table — (DOCX) [file pone.0176967.s001.docx]

S1 Table

|  |  | **Total population** | | **Non-bleeding patients** | | **Bleeding patients** | | |
| --- | --- | --- | --- | --- | --- | --- | --- | --- |
|  |  | **Without co-medication (n = 97)** | **With co-medication (n = 29)** | **Without co-medication (n = 84)** | **With co-medication (n = 17)** | **Without co-medication (n = 13)** | **With co-medication (n = 12)** | |
| **ETP (nM.min)** | **Median** | 242.2 | 266.0 | 255.6 | 266.0 | 182.5 | 231.4 | |
|  | **IQR** | 182.0-332.6 | 178.7-330.4 | 189.6-341.3 | 217.6-351.3 | 152.4-197.4 | 163.7-302.5 | |
|  | **Confidence interval** | 242.7-287.9 | 229.9-322.8 | 252.7-302.3 | 233.9-368.9 | 154.6-218.9 | 175.5-306.3 | |
|  | **P value** | 0.7453 | | 0.5892 | | 0.3695 | | |
| **Peak (nM)** | **Median** | 36.31 | 34.9 | 39.6 | 34.9 | 22.4 | | 32.2 |
|  | **IQR** | 22.3-50.5 | 25.8-57.1 | 23.5-52.0 | 30.8-59.1 | 19.6-34.2 | | 18.3-57.3 |
|  | **Confidence interval** | 35.4-44.5 | 34.1-54.1 | 36.8-46.9 | 33.0-62.1 | 18.9-36.5 | | 24.4-54.2 |
|  | **P value** | 0.4477 | | 0.4595 | | 0.2422 | | |

**The use of co-medications did not influence whole blood ETP and peak.**

IQR, interquartile range
