# Supplementary material for: Prediction of bleeding risk in patients taking vitamin K antagonists using thrombin generation testing
Source: PLoS One. 2017 May 4;12(5):e0176967. doi: 10.1371/journal.pone.0176967 (PMC5417600; doi:10.1371/journal.pone.0176967)
Supplement: S2 Table — (DOCX) [file pone.0176967.s002.docx]

S2 Table

| R-value | **ETP (nM.min)** | **Peak (nM)** | **Lag time (min)** | **Time-to-peak (min)** |
| --- | --- | --- | --- | --- |
| **PRP** | 0.593 | 0.651 | 0.300 | 0.449 |
|  | p = 0.000 | p = 0.000 | p = 0.001 | p = 0.000 |
| **PPP (5 pM TF)** | 0.675 | 0.815 | 0.426 | 0.510 |
|  | p = 0.000 | p = 0.000 | p = 0.000 | p = 0.000 |
| **PPP (1 pM TF)** | 0.613 | 0.767 | 0.252 | 0.506 |
|  | p = 0.000 | p = 0.000 | p = 0.004 | p = 0.000 |

**Correlation of whole blood CAT with plasma CAT.**

PRP, platelet rich plasma; PPP, platelet poor plasma; TF, tissue factor; ETP, endogenous thrombin potential
