# Supplementary material for: Prediction of bleeding risk in patients taking vitamin K antagonists using thrombin generation testing
Source: PLoS One. 2017 May 4;12(5):e0176967. doi: 10.1371/journal.pone.0176967 (PMC5417600; doi:10.1371/journal.pone.0176967)
Supplement: S3 Table — (DOCX) [file pone.0176967.s003.docx]

S3 Table

| R-value | **ETP (nM.min)** | **Peak (nM)** | **Lag time (min)** | **Time-to-peak (min)** |
| --- | --- | --- | --- | --- |
| **Whole blood** | -0.456 | -0.720 | 0.504 | 0.622 |
|  | p = 0.000 | p = 0.000 | p = 0.000 | p = 0.000 |
| **PRP** | -0.626 | -0.653 | 0.581 | 0.605 |
|  | p = 0.000 | p = 0.000 | p = 0.000 | p = 0.000 |
| **PPP (5 pM TF)** | -0.748 | -0.782 | 0.466 | 0.489 |
|  | p = 0.000 | p = 0.000 | p = 0.000 | p = 0.000 |
| **PPP (1 pM TF)** | -0.784 | -0.768 | 0.384 | 0.377 |
|  | p = 0.000 | p = 0.000 | p = 0.000 | p = 0.000 |

**Correlation of INR with CAT parameters.**

PRP, platelet rich plasma; PPP, platelet poor plasma; TF, tissue factor; ETP, endogenous thrombin potential
