# Supplementary material for: Prediction of bleeding risk in patients taking vitamin K antagonists using thrombin generation testing
Source: PLoS One. 2017 May 4;12(5):e0176967. doi: 10.1371/journal.pone.0176967 (PMC5417600; doi:10.1371/journal.pone.0176967)
Supplement: S4 Table — (DOCX) [file pone.0176967.s004.docx]

S4 Table

|  | **Prothrombin** | | **Factor V** | | **Factor VII** | | **Factor VIII** | | **Factor IX** | | **Factor X** | |
| --- | --- | --- | --- | --- | --- | --- | --- | --- | --- | --- | --- | --- |
|  | Bleeding | Non-bleeding | Bleeding | Non-bleeding | Bleeding | Non-bleeding | Bleeding | Non-bleeding | Bleeding | Non-bleeding | Bleeding | Non-bleeding |
| **n** | 26 | 129 | 26 | 128 | 26 | 127 | 17 | 105 | 24 | 124 | 26 | 128 |
| **Median** | 19 | 23 | 101.5 | 104 | 17 | 15 | 174 | 179 | 33 | 36 | 11 | 13 |
| **[IQR 25%-75%]** | [15.8-27.3] | [16.5-29] | [89.8-115.3] | [91-115] | [12-24.3] | [10-23] | [135-234] | [141-218.5] | [24.3-41.8] | [26-46.8] | [8-14.8] | [9-16] |
| **CI** | 18.4-25.1 | 22.4-26.0 | 95.1-112.1 | 101.0-107.4 | 14.2-25.0 | 16.3-20.8 | 152.0-222.- | 173.2-195.7 | 29.3-42.9 | 36.1-42.9 | 10.3-14.7 | 12.9-15.4 |
| **P-value** | 0.304 |  | 0.806 |  | 0.502 |  | 0.968 |  | 0.357 |  | 0.197 |  |

|  | **Protein C** | | **Protein S** | | **Antithrombin** | |
| --- | --- | --- | --- | --- | --- | --- |
|  | Bleeding | Non-bleeding | Bleeding | Non-bleeding | Bleeding | Non-bleeding |
| **n** | 23 | 101 | 15 | 99 | 26 | 129 |
| **Median** | 10 | 11 | 35 | 36 | 112 | 108 |
| **[IQR 25%-75%]** | [5-23] | [5.3-23] | [25-40] | [27-42] | [104.5-118.8] | [102-116] |
| **CI** | 9.1-21.6 | 13.0-19.2 | 26.9-41.3 | 34.3-39.7 | 107.8-116.4 | 106.9-111.4 |
| **P-value** | 0.953 |  | 0.443 |  | 0.235 |  |

**Medians with interquartile ranges of coagulation factor determinations.**

IQR, interquartile range; CI, confidence interval
