# Supplementary material for: Prediction of bleeding risk in patients taking vitamin K antagonists using thrombin generation testing
Source: PLoS One. 2017 May 4;12(5):e0176967. doi: 10.1371/journal.pone.0176967 (PMC5417600; doi:10.1371/journal.pone.0176967)
Supplement: S1 Supporting information — (DOCX) [file pone.0176967.s005.docx]

S1 Supporting information, Table 1

|  | ***Non-bleeding*** | | | | | ***Bleeding*** | | | | |  |
| --- | --- | --- | --- | --- | --- | --- | --- | --- | --- | --- | --- |
|  | *n* | *median* | *IQR* | *min-max* | *CI* | *n* | *median* | *IQR* | *min-max* | *CI* | *p-value* |
| INR | 103 | 2.9 | 2.3-3.7 | 1.1-5.7 | 2.8-3.1 | 26 | 3.0 | 2.3-3.5 | 1.4-5.1 | 2.6-3.3 | 0.874 |
| Hematocrit | 103 | 0.38 | 0.36-0.42 | 0.24-0.47 | 0.38-0.40 | 26 | 0.38 | 0.35-0.40 | 0.25-0.46 | 0.35-0.39 | 0.232 |
| Hemoglobin | 103 | 7.6 | 7.1-8.2 | 4.6-9.9 | 7.5-7.8 | 26 | 7.4 | 6.6-7.9 | 5.2-9.1 | 6.9-7.7 | 0.112 |
| Fibrinogen | 102 | 3.9 | 3.4-4.5 | 2.4-6.5 | 3.8-4.2 | 26 | 3.9 | 3.4-4.8 | 2.5-5.5 | 3.8-4.4 | 0.540 |

**Medians with ranges of parameters which are known to be related with bleeding.**

INR, international normalized ratio; IQR, interquartile range; CI, confidence interval

S1 Supporting information, Figure 1





**S1 Fig 1. Hematocrit, hemoglobin, international normalized ratio (INR) and fibrinogen determinations in patients with and without bleeding symptoms.** Determinations of (A) hematocrit (p = 0.232), (B) hemoglobin (p = 0.112), (C) INR (p = 0.874) and (D) fibrinogen (p = 0.540) in patients with bleeding compared to without bleeding.
